# Sensitivity, specificity, and regression‐based norms for the digital version of the Neurocognitive Frailty Index

**DOI:** 10.1002/alz.090030

**Published:** 2025-01-09

**Authors:** Sarah Pakzad, Paul Bourque, Daniel Saucier

**Affiliations:** ^1^ Université de Moncton, Moncton, NB Canada

## Abstract

**Background:**

Integrating technology in the cognitive assessment process could help with dementia care and management (Astell et al., 2019). The aim of this research was to assess the sensitivity and specificity, as well as establish regression‐based norms, for the digital version of the Neurocognitive Frailty Index (NFI, Pakzad et al., 2017).

**Method:**

The digital version of the NFI was administered alongside the Montreal Cognitive Assessment (MoCA) on 238 people aged 50+ recruited from various communities across the province of New Brunswick, Canada. Agreement for mild cognitive impairment (MCI) between the digital NFI and the MoCA were analyzed. Normative data for the digital NFI was also derived from the study sample by modeling the distribution of the raw test scores conditional upon the norm‐predictors (age, sex, and level of education).

**Result:**

Mean digital NFI score and standard deviation was 56.3±5.91, while mean MoCA score and standard deviation was 26.4±2.89. Area under the curve (AUC) for detecting MCI with the digital NFI in comparison to recommended MoCA cut‐off of normal ≥ 26 was 0.89 (0.84‐0.93 95% confidence interval). The optimal digital NFI cut‐off score for MCI was normal ≥ 57 with sensitivity of 0.92 and specificity of 0.78. All predictors contributed to establishing regression‐based norms for the digital NFI as to calculate expected scores given the age, sex, and level of education of an individual.

**Conclusion:**

Age, sex, and education should be considered when using the NFI assessment tool. This will be facilitated by the regression‐based calculator created from these results that will be imbedded into the digital version of the NFI. This will provide percentiles for a subject’s NFI score within a personalized report that is auto generated upon test completion within the online digital NFI platform. The study results attest to the robustness of the digital version of the NFI as a measure with significant sensitivity and specificity.

